# Protective effects of shikimic acid against thioacetamide-induced hepatic fibrosis: role of Nrf2/NF-κB signaling pathways

**DOI:** 10.3389/fphar.2025.1609711

**Published:** 2025-05-30

**Authors:** Ashraf Albrakati

**Affiliations:** Department of Anatomy, College of Medicine, Taif University, Taif, Saudi Arabia

**Keywords:** shikimic acid, thioacetamide, liver fibrosis, oxidative stress, inflammation, apoptosis

## Abstract

**Background:**

Liver fibrosis is a chronic condition marked by scar tissue accumulation in the liver that impairs function. Shikimic acid (SA) has demonstrated significant antioxidant and anti-inflammatory properties and is a hydroaromatic natural product found in various plant sources, including star anise. Thioacetamide (TAA) is commonly used as an experimental model to induce liver fibrosis. This study was designed to investigate the protective effects of SA against TAA-induced liver fibrosis in rats.

**Methods:**

Fifty male Wistar rats were divided into five groups (n = 10): control, TAA (200 mg/kg), SA (50 mg/kg), and two combination groups receiving TAA plus SA (50 and 100 mg/kg). Treatments were administered intraperitoneally for 6 weeks. Liver function, oxidative stress markers, inflammatory mediators, apoptotic proteins, fibrogenic factors, and histopathology were assessed.

**Results:**

SA treatment significantly attenuated liver fibrosis induced by TAA through improved liver enzymes, including alkaline phosphatase (ALP) and aspartate transaminase (AST), serum alanine transaminase (ALT) and antioxidant parameters, including catalase (CAT), glutathione peroxidase (GPx), glutathione reductase (GR), and superoxide dismutase (SOD). SA-enhanced nuclear factor erythroid 2-related factor-2 (Nrf2) expression while reducing oxidative stress markers, including nitric oxide (NO), malondialdehyde (MDA) and reduced glutathione (GSH), and inflammatory mediators, including tumour necrosis factor-alpha (TNF-α), interleukin-1 beta (IL-1β) and nuclear factor kappa-light-chain-enhancer of activated B-cells (NF-κB). Furthermore, SA demonstrated anti-apoptotic effects by modulating Bax, Bcl2, and caspase-3 levels and exhibited anti-fibrotic properties through suppression of transforming growth factor beta (TGF-β) and collagen type I alpha-1 chain (COL1A1) expressions. Notably, SA improved histopathological alterations with a slight presence of fibroplasia. These protective effects were more pronounced at the higher dose (100 mg/kg).

**Conclusion:**

SA effectively protected against TAA-induced liver fibrosis through multiple mechanisms, including the normalisation of liver enzymes, enhancement of antioxidant defences via Nrf2 activation, suppression of inflammatory mediators, and modulation of apoptotic and fibrogenic pathways.

## 1 Introduction

Understanding the molecular mechanisms and pathways underlying liver fibrosis progression is crucial for developing effective therapeutic strategies, as thioacetamide (TAA) knowledge enables the identification of potential therapeutic targets and the development of novel treatments that can prevent or reverse hepatic damage and fibrotic changes.

TAA, a sulfur-containing compound widely used as an experimental model for inducing liver fibrosis and cirrhosis, exerts its hepatotoxic effects through metabolic activation by cytochrome P450 enzymes in the liver. The resulting toxic metabolite, TAA-S-oxide, triggers a complex pathological cascade primarily through the generation of reactive oxygen species (ROS) and subsequent oxidative stress in hepatic tissue (Ezhilarasan, 2023). TAA oxidative insult triggers enhanced nuclear factor erythroid 2-related factor-2 (Nrf2) translocation to the nucleus, where it binds to antioxidant response elements to regulate key antioxidant enzymes, including glutathione peroxidase (GPx), superoxide dismutase (SOD), and catalase (CAT), that work synergistically to neutralise ROS (Carmo de Carvalho e Martins et al., 2022; Bardallo et al., 2022; Jomova et al., 2024). Concurrently, TAA activates Kupffer cells to release pro-inflammatory cytokines including interleukin-1 beta (IL-1β) and tumour necrosis factor-alpha (TNF-α), initiating an inflammatory cascade (Dwivedi and Jena, 2022). The combined effects of oxidative stress and inflammatory mediators activate hepatic stellate cells, promoting their transformation into myofibroblasts that produce excessive collagen, ultimately leading to liver fibrosis (Salguero Palacios et al., 2008) and potential progression to cirrhosis if left untreated (Poynard et al., 1997).

The increased oxidative stress and inflammation in hepatic tissue promotes apoptotic cell death through multiple interconnected pathways (Li et al., 2016). Under oxidative stress conditions, the balance between pro-apoptotic Bcl-2-associated X protein (Bax) and anti-apoptotic B-cell lymphoma 2 (Bcl-2) proteins becomes disrupted, favoring increased Bax expression (Roufayel, 2016). Bax protein translocation to the mitochondria leads to the release of cytochrome c, which subsequently activates caspase cascades, particularly cysteine-aspartic acid protease 3 (caspase-3), the main executioner of apoptosis (Kirkland and Franklin, 2003).

Shikimic acid (SA) (3,4,5-trihydroxy-1-cyclohexene-1-carboxylic acid) is a crucial natural compound predominantly found in star anise (Illicium verum), with significant quantities also present in sweetgum fruit (Liquidambar styraciflua), pine needles (Pinus spp.), and various other plant species (Estevez and Estevez, 2012; Candeias et al., 2018). TAA compound plays a vital role in the biosynthesis of aromatic amino acids in plants and microorganisms through the shikimate pathway, and has gained considerable attention as a key starting material for the synthesis of various pharmaceutical compounds (Santos-Sánchez et al., 2019). Its pharmacological derivatives possess a wide range of bioactivities, including antioxidant (Sun et al., 2016), antiviral, anti-inflammatory (Gandhi et al., 2023), and antibacterial properties, combined with low toxicity, making them promising candidates for therapeutic applications across various organ systems (Quiroz et al., 2014).

Previous studies have demonstrated the therapeutic efficacy of SA in various pathological conditions, including cardiovascular diseases (Gu et al., 2024), viral disease (Xing et al., 2013), renal injuries (Lee et al., 2020), and neural disease (Bao et al., 2023). Therefore, TAA study was designed to investigate the potential protective effects of SA against TAA-induced liver fibrosis in rats by examining various biochemical, molecular, and histological parameters.

## 2 Materials and methods

TAA and SA were purchased from Sigma-Aldrich, St. Louis, MO, United States.

### 2.1 Animals and experimental design

Male Wistar rats (weighing 180–200 g) were obtained from the King Fahd Center for Genetic Research, King Abdulaziz University, Kingdom of Saudi Arabia. Animals were housed under standard laboratory conditions (temperature 22°C ± 2°C, 12-h light/dark cycle, relative humidity 55% ± 5%) with free access to standard pellet diet and water *ad libitum*. After 1 week of acclimatization, rats were randomly divided into five groups (n = 10 per group).

- Group I (Control): Rats received normal saline for 6 weeks.
- Group II (SA): According to Eissa et al. (2018), rats were treated with SA (50 mg/kg, i. p., daily for 6 weeks).
- Group III (TAA): According to Al-Malki (2019), rats were treated with TAA (200 mg/kg, ip) twice a week for 6 weeks.
- Group IV (SA-50+TAA): Rats were treated with TAA (200 mg/kg, ip) twice a week for 6 weeks. Rats were pretreated with SA (50 mg/kg)1 h before treatment with TAA for 6 weeks.
- Group V (SA-100+TAA): TAA + SA (200 mg/kg+100 mg/kg). Rats were pretreated with SA 1 h before treatment with TAA for 6 weeks.

TAA was prepared fresh daily by dissolving in sterile 0.9% saline, while SA was suspended in 0.5% carboxymethylcellulose (CMC) solution.

### 2.2 Sample collection and preparation

At the end of the experimental period, rats were fasted overnight and euthanized using thiopental sodium (100 mg/kg, intraperitoneally), in accordance with internationally accepted guidelines for the care and use of laboratory animals. Blood samples were collected via cardiac puncture, allowed to clot, and centrifuged at 3,000 rpm for 15 min to obtain serum. Liver tissues were immediately excised, rinsed with ice-cold saline, and divided into portions for various analyses. Samples for biochemical assays were stored at −80°C until use. A portion of the liver was fixed in 4% neutral-buffered formalin for histopathological examination.

### 2.3 Biochemical analyses

#### 2.3.1 Liver function tests

Serum alanine transaminase (ALT, Sigma-Aldrich, St. Louis, MO, United States), alkaline phosphatase (ALP, Sigma-Aldrich, St. Louis, MO, United States), and aspartate transaminase (AST, Sigma-Aldrich, St. Louis, MO, United States) activities were assessed according to Reitman and Frankel (1957). Liver function enzymes (ALT, AST, and ALP) were measured in serum/plasma samples.

#### 2.3.2 Oxidative stress and antioxidants parameters

Oxidative stress markers and antioxidant activities were assessed in liver tissue through various spectrophotometric methods. The quantification of malondialdehyde (MDA, Abcam Limited, Cambridge Biomedical Campus, Cambridge, CB2 0AX, UK) was conducted as a measure of lipid peroxidation, using the procedure established by Ohkawa et al. (1979). The level of hepatic nitric oxide (NO, Sigma-Aldrich, St. Louis, MO, United States) was assessed using the technique described by Robbins et al. (1996). The concentration of reduced glutathione (GSH, Thermo Fisher Scientific Inc. 168 Third Avenue, Waltham, MA 02451, United States) in the liver homogenates was determined using the method outlined by Ellman (1959).

The activity of hepatic SOD (Sigma-Aldrich, St. Louis, MO, United States) was assessed using the technique described by Nishikimi et al. (1972). The level of hepatic glutathione reductase (GR, Sigma-Aldrich, St. Louis, MO, United States) was assessed using the technique described by Moron et al. (1979). CAT (Sigma-Aldrich, St. Louis, MO, United States) activity was determined using the technique described by Aebi (1984). GPx (Sigma-Aldrich, St. Louis, MO, United States) activity was assessed using the technique described by Paglia and Valentine (1967). The protein content of the liver was assessed using the methodology established by Lowry et al. (1951). Nuclear factor erythroid 2-related factor 2 (Nrf2, Elabscience Biotechnology Inc., United States) levels were quantified using a specific ELISA kit according to the protocols.

#### 2.3.3 Inflammatory markers

Liver tissue levels of inflammatory mediators including TNF-α, IL-1β, and nuclear factor kappa-light-chain-enhancer of activated B-cells (NF-κB) were measured using specific ELISA kits (Elabscience Biotechnology Inc., United States) according to manufacturer’s protocols.

#### 2.3.4 Apoptotic markers

Liver tissue levels of Bcl-2 and Bax were measured using ELISA kits (BioVision, Inc., United States). Caspase-3 activity was assessed using a colorimetric kit (Sigma-Aldrich, St. Louis, MO, United States). All assays were conducted according to manufacturer’s instructions.

#### 2.3.5 Gene expression analysis

The total RNA was extracted from liver tissue using the RNeasy Plus Mini-kit (Qiagen, Valencia, CA, United States). First-stranded cDNA was synthesized using a cDNA synthesis kit (Bio-Rad, CA, United States), then amplified in three technical replicates using Power SYBR Green (Life Technologies, CA, United States) and measured using an Applied Biosystems 7,500 instrument (Roche Molecular Systems, Inc., Foster City, CA, United States).

The Quantitative real-time PCR (Q-RT-PCR) conditions were set at 95°C for 4 min, followed by 40 cycles of 94°C for 60 s and 55°C for 60 s, with a final extension at 72°C for 10 min. After amplification, cycle numbers at the linear amplification threshold (Ct) for the housekeeping gene β-actin were determined for each sample, and relative gene expression was determined using the comparative Ct method (Pfaffl, 2001).

Q-RT-PCR primers for Caspase-3, IL-1β, Nrf2, collagen type I alpha-1 chain (COL1A1), transforming growth factor beta (TGF-β), and β-actin were designed using the Primer-Blast program provided by the National Center for Biotechnology Information (NCBI) and synthesized by Jena Bioscience GmbH (Jena, Germany). Table 1 presents the primer sequences and accession numbers for the genes examined in TAAs study. Relative fold changes in gene expression were calculated using the ΔΔCt method after normalization to the housekeeping gene β-actin.

**TABLE 1:** Primers sqRT-PCR analysis.

Name	Accession number	Sense (5′-3′)	Antisense (5′-3′)
β-actin	NM_031144.3	TCT​TCC​AGC​CTT​CCT​TCC​TG	CAC​ACA​GAG​TAC​TTG​CGC​TC
Caspase-3	NM_012922.2	GGC​CGA​CTT​CCT​GTA​TGC​TT	CGT​ACA​GTT​TCA​GCA​TGG​CG
IL-1β	NM_204524.2	CGA​CAT​CAA​CCA​GAA​GTG​CTT	GTC​CAG​GCG​GTA​GAA​GAT​GA
Nrf2	NM_031789.2	CAG​CAT​GAT​GGA​CTT​GGA​ATT​G	GCA​AGC​GAC​TCA​TGG​TCA​TC
COL1a1	NM_021578.2	ACC​AAC​TAC​TGC​TTC​AGC​TCC​ACA	TGT​ACT​GTG​TGT​CCA​GGC​TCC​AAA
TGF-β1	NM_053304.1	ATC​AGC​CCA​AAC​CCC​AAG​GAG	CGC​AGG​AAG​GTC​AGC​TGG​ATA​G

#### 2.3.6 Histopathological examination

Liver tissue samples were fixed in 4% neutral buffered formalin for 24 h, dehydrated through ascending grades of ethanol (70%-80%-90%–100%, 2 min in each), cleared in xylene (twice, 5 min in each), and embedded in paraffin wax. Sections of 5 μm tackiness were cut using a rotary microtome (Leica RM2235, Germany) and mounted on glass slides (Drury and Wallington, 1980; Ahmad et al., 2022).

##### 2.3.6.1 Hematoxylin and eosin staining method

Sections were deparaffinized in xylene and rehydrated through descending ethanol grades. Nuclei were stained with Mayer’s hematoxylin for 5 min, followed by differentiation in 1% acid alcohol and bluing in Scott’s tap water substitute. The sections were counterstained with eosin Y for 1–2 min, dehydrated, cleared in xylene, and mounted with DPX (Cardiff et al., 2014; Che et al., 2014). Stained sections were examined under a light microscope (Olympus BX51, Japan) and photographed using a digital camera (Olympus DP27, Japan).

##### 2.3.6.2 Masson’s trichrome staining method

Deparaffinized and rehydrated sections were treated with Bouin’s solution at 56°C for 1 h and rinsed in tap water. Nuclei were stained with Weigert’s iron hematoxylin for 10 min, followed by staining with Biebrich scarlet-acid fuchsin to highlight muscle and cytoplasm. Collagen fibers were selectively stained with Aniline Blue for 5 min after differentiation in phosphomolybdic acid. Sections were briefly treated with 1% acetic acid, dehydrated, cleared, and mounted for examination under a light microscope (Garvey, 1984).

#### 2.3.7 Statistical analysis

Data were expressed as mean ± standard error of mean (SEM). Statistical analysis was performed using GraphPad Prism software (version 8.0). Differences between groups were analyzed using one-way analysis of variance (ANOVA) followed by Tukey’s post-hoc test. P values <0.05 were considered statistically significant.

## 3 Results

### 3.1 Liver enzyme level tests

The biochemical analysis of liver enzymes (ALP, AST, and ALT) revealed significant changes across treatment groups. Control rats showed baseline levels of ALP (100 ± 12 U/L), AST (42 ± 5 U/L), and ALT (90 ± 10 U/L), which remained stable with SA alone treatment (p > 0.05). TAA administration significantly elevated liver enzymes (p < 0.05): ALP increased by 65% (165 ± 12 U/L), AST by 114% (90 ± 10 U/L), and ALT by 61% (145 ± 10 U/L) compared to controls.

**FIGURE 1:**
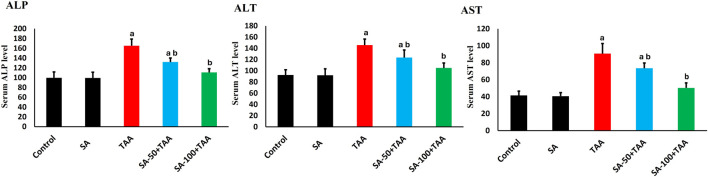
Effects of SA on serum liver enzymes (ALP, AST, and ALT) in TAA-induced liver fibrosis in rats. Values are expressed as mean ± SD (n = 10). ^a^ and ^b^ denote significant differences (p < 0.05) compared with the untreated control and TAA-injected groups, respectively.

SA demonstrated dose-dependent protective effects. At 50 mg, enzyme levels were partially reduced (p < 0.05): ALP (132 ± 8 U/L), AST (72 ± 8 U/L), and ALT (122 ± 12 U/L). The 100 mg dose showed superior protection (p < 0.05): ALP reduced to 112 ± 8 U/L, AST to 50 ± 5 U/L, and ALT to 105 ± 8 U/L. SA exerted dose-dependent hepatoprotection against TAA-induced liver injury, with the 100 mg/kg dose most effectively normalizing ALP, AST, and ALT levels (Figure 1).

### 3.2 Oxidative stress parameters analysis

Analysis of oxidative stress markers (GSH, NO, MDA) revealed significant changes across treatment groups. Control rats showed baseline levels of GSH (1.5 ± 0.1 mg/dl tissue), NO (105 ± 10 μmol/g tissue), and MDA (110 ± 15 nmol/g tissue), which remained unchanged with SA alone treatment (p > 0.05). TAA administration significantly altered these markers (p < 0.05): GSH decreased by 67% (0.5 ± 0.2 mg/dl tissue), while NO and MDA increased by 52% (160 ± 12 μmol/g tissue) and 109% (230 ± 20 nmol/g tissue) respectively, indicating severe oxidative stress and membrane damage.

**FIGURE 2:**
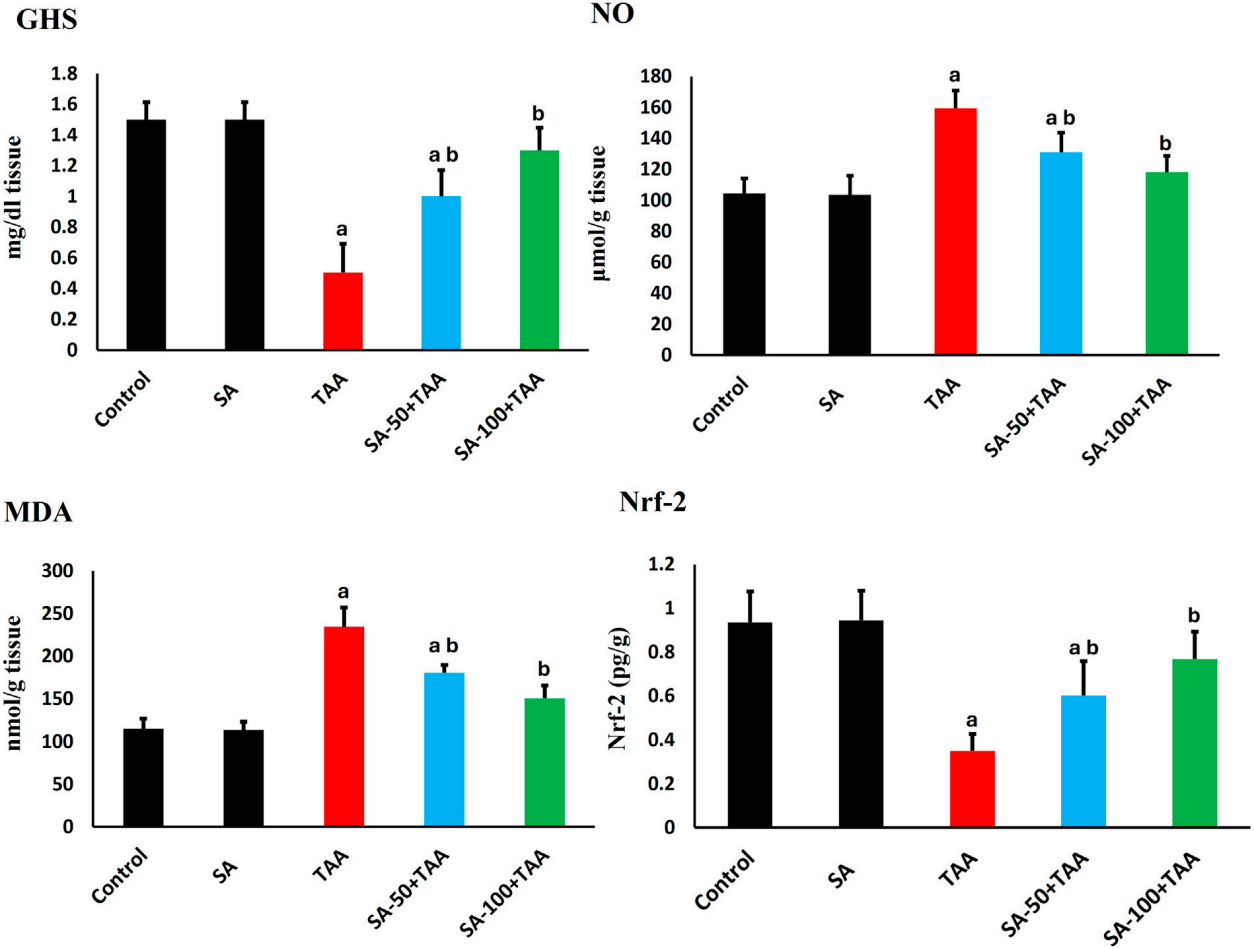
Effects of SA on hepatic GSH, NO, MDA, and Nrf-2 levels in TAA-induced liver fibrosis in rats. Values are expressed as mean ± SD (n = 10). ^a^ and ^b^ denote significant differences (p < 0.05) compared with the untreated control and TAA-injected groups, respectively.

SA demonstrated dose-dependent protection, with 100 mg showing superior effects (p < 0.05): GSH increased to 1.3 ± 0.1 mg/dl tissue, NO decreased to 120 ± 10 μmol/g tissue, and MDA reduced to 150 ± 15 nmol/g tissue. Analysis of Nrf-2 showed significant alterations, with TAA reducing expression by 60% (0.4 ± 0.15 fold-change, p < 0.05). SA co-treatment restored Nrf-2 levels dose-dependently, with 100 mg enhancing levels to 1.7 ± 0.2 fold-change (p < 0.05), indicating robust activation of antioxidant responses (Figure 2). These results demonstrate that SA restores redox homeostasis and suppresses lipid peroxidation following TAA exposure, underscoring its potent antioxidative and hepatoprotective actions.

### 3.3 Analysis of antioxidant activities

Analysis of antioxidant enzymes revealed significant changes across treatment groups. In control rats, baseline activities showed normal physiological values: CAT (1.6 ± 0.2 U/mg protein), GPx (1.2 ± 0.1 pg/mg protein), GR (0.8 ± 0.1 μmol/mg protein), and SOD (6.0 ± 0.7 U/mg protein). Treatment with SA alone maintained these enzyme activities at comparable levels, demonstrating no adverse effects on the baseline antioxidant defense system.

TAA administration induced significant reductions (p < 0.05) in all antioxidant enzymes. CAT activity decreased by 63% to 0.6 ± 0.2 U/mg protein, GPx activity reduced by 58% to 0.5 ± 0.3 pg/mg protein, GR activity declined by 50% to 0.4 ± 0.15 μmol/mg protein, and SOD activity decreased by 45% to 3.3 ± 0.7 U/mg protein, indicating comprehensive impairment of cellular antioxidant defenses.

**FIGURE 3:**
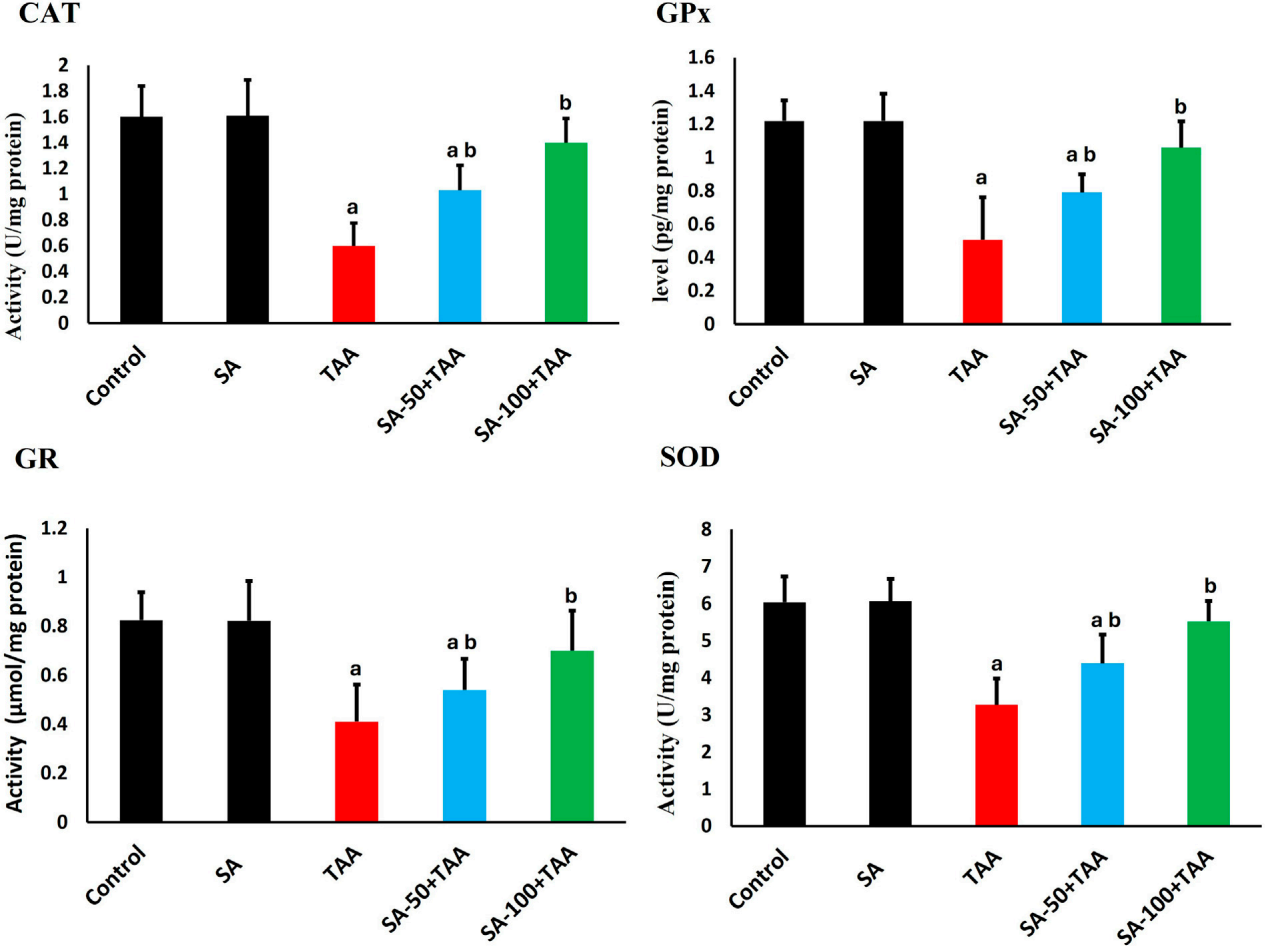
Effects of SA on hepatic antioxidant enzymes (CAT, GPx, GR, and SOD) in TAA-induced liver fibrosis in rats. Values are expressed as mean ± SD (n = 10). ^a^ and ^b^ denote significant differences (p < 0.05) compared with the untreated control and TAA-injected groups, respectively.

SA co-treatment demonstrated dose-dependent improvements. The higher dose of 100 mg showed significant restoration (p < 0.05), with CAT increasing to 1.4 ± 0.2 U/mg protein, GPx to 1.1 ± 0.1 pg/mg protein, GR to 0.7 ± 0.15 μmol/mg protein, and SOD to 5.5 ± 0.5 U/mg protein (Figure 3).

### 3.4 Analysis of inflammatory markers

**FIGURE 4:**
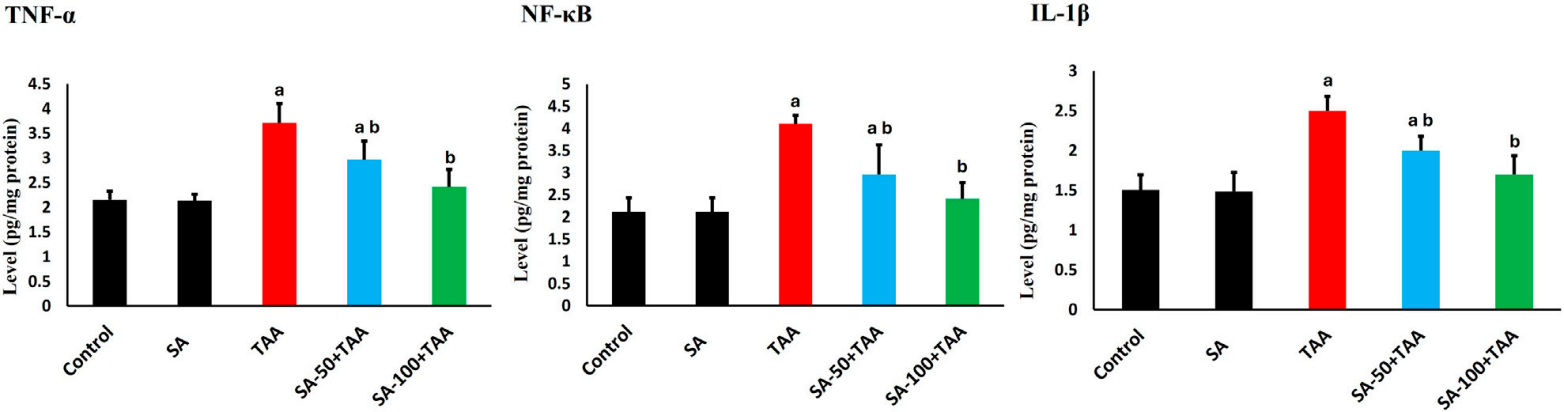
Effects of SA on hepatic TNF-α, IL-1β, and NF-κB levels in TAA-induced liver fibrosis in rats. Values are expressed as mean ± SD (n = 10). ^a^ and ^b^ denote significant differences (p < 0.05) compared with the untreated control and TAA-injected groups, respectively.

The analysis of inflammatory markers (TNF-α, IL-1β, and NF-κB) revealed significant changes across treatment groups. Control rats showed baseline protein levels of TNF-α (2.1 ± 0.2 pg/mg protein), IL-1β (1.5 ± 0.2 pg/mg protein), and NF-κB (2.1 ± 0.3 pg/mg protein), which remained unchanged with SA alone treatment (p > 0.05). TAA administration significantly elevated these markers (p < 0.05): TNF-α by 76% (3.7 ± 0.3 pg/mg protein), IL-1β by 67% (2.5 ± 0.2 pg/mg protein), and NF-κB by 95% (4.1 ± 0.2 pg/mg protein). SA showed dose-dependent effects, with 50 mg showing moderate improvement (p < 0.05) and 100 mg being more effective (p < 0.05) in normalizing levels: TNF-α (2.3 ± 0.3 pg/mg protein), IL-1β (1.7 ± 0.2 pg/mg protein), and NF-κB (2.3 ± 0.3 pg/mg protein). These results underscore SA’s dose-dependent anti-inflammatory efficacy, effectively suppressing TAA-induced elevations in TNF-α, IL-1β, and NF-κB, thereby enhancing its hepatoprotective properties (Figure 4).

### 3.5 Analysis of apoptotic markers

The analysis of apoptotic markers (Bax, Bcl-2, and Caspase-3) demonstrated significant changes across treatment groups. Control rats showed baseline levels of Bax (2.4 ± 0.2 ng/mg), Bcl-2 (3.7 ± 0.4 ng/mg), and Caspase-3 (2.4 ± 0.2 ng/mg), which remained stable with SA alone treatment (p > 0.05).

**FIGURE 5:**
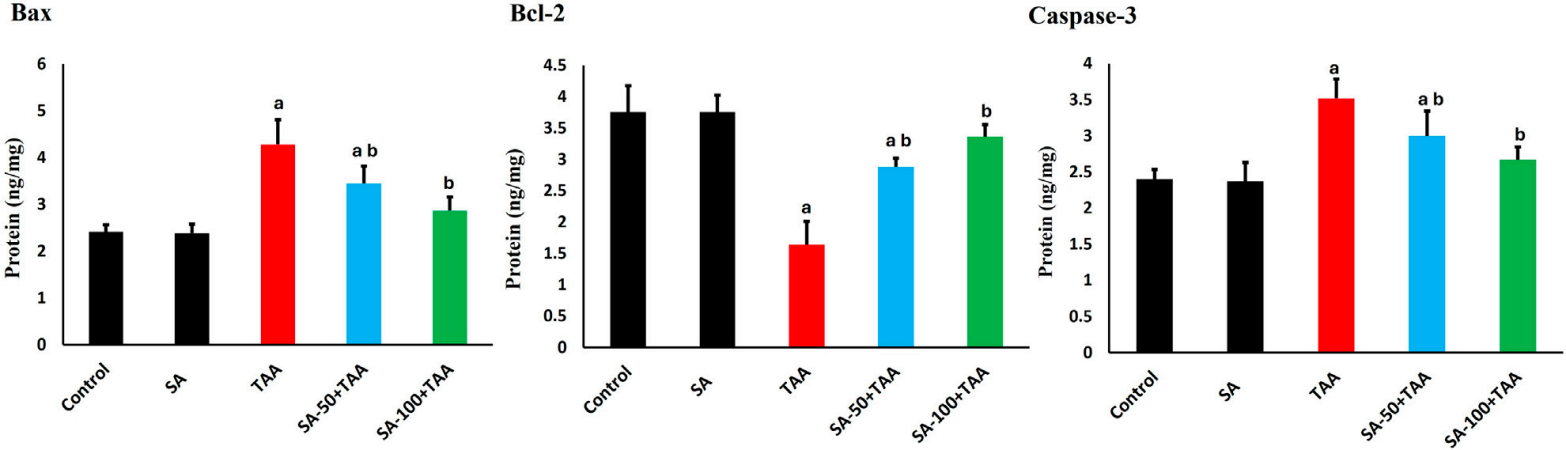
Effects of SA on hepatic Bax, Bcl-2, and Caspase-3 protein levels in TAA-induced liver fibrosis in rats. Values are expressed as mean ± SD (n = 10). ^a^ and ^b^ denote significant differences (p < 0.05) compared with the untreated control and TAA-injected groups, respectively.

TAA administration significantly increased pro-apoptotic protein Bax by 75% (4.2 ± 0.5 ng/mg) and Caspase-3 by 46% (3.5 ± 0.3 ng/mg), while decreasing the anti-apoptotic Bcl-2 by 57% (1.6 ± 0.4 ng/mg) compared to control (p < 0.05). SA showed dose-dependent protective effects, with 50 mg showing moderate improvement (p < 0.05): Bax (3.5 ± 0.3 ng/mg), Bcl-2 (2.9 ± 0.2 ng/mg), and Caspase-3 (3.0 ± 0.3 ng/mg), while 100 mg demonstrated superior protection (p < 0.05): Bax (2.8 ± 0.3 ng/mg), Bcl-2 (3.3 ± 0.2 ng/mg), and Caspase-3 (2.6 ± 0.2 ng/mg). SA displayed dose-dependent anti-apoptotic effects in TAA-treated rats, with the 100 mg/kg dose most effectively reducing pro-apoptotic markers Bax and Caspase-3 while restoring the anti-apoptotic Bcl-2 levels (Figure 5).

### 3.6 Molecular analysis of antioxidant, apoptotic, and inflammatory gene expression

The analysis of gene expression levels for key regulatory markers (Nrf-2, Caspase-3, and IL-1β) revealed significant changes across treatment groups. Control rats showed normalized baseline mRNA expression (1.0 ± 0.2 fold-change) for all markers, which remained stable with SA alone treatment (p > 0.05).

TAA administration significantly altered gene expression patterns (p < 0.05): Nrf-2 decreased by 60% (0.4 ± 0.1 fold-change), while Caspase-3 and IL-1β increased by 120% (2.2 ± 0.2 fold-change) and 100% (2.0 ± 0.3 fold-change) respectively, indicating suppressed antioxidant responses and enhanced apoptotic and inflammatory signaling.

**FIGURE 6:**
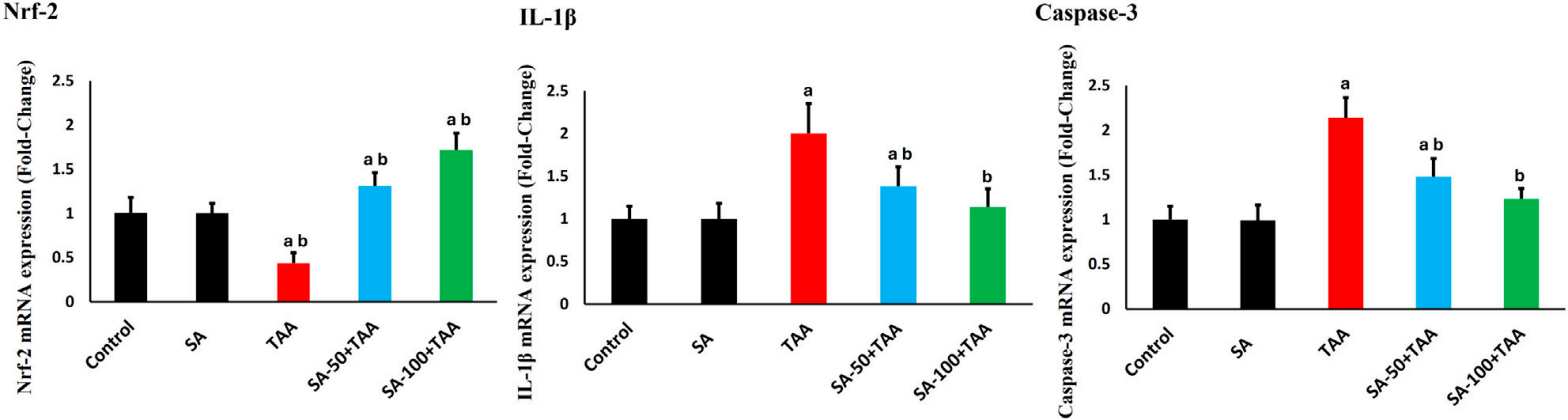
Effects of SA on hepatic mRNA expression levels of Nrf-2, Caspase-3, and IL-1β in TAA-induced liver fibrosis in rats. Values are expressed as mean ± SD (n = 10). ^a^ and ^b^ denote significant differences (p < 0.05) compared with the untreated control and TAA-injected groups, respectively.

SA demonstrated dose-dependent modulatory effects. The higher dose (100 mg) showed more pronounced effects (p < 0.05): Nrf-2 expression increased to 1.7 ± 0.2 fold-change, while Caspase-3 and IL-1β levels decreased to 1.2 ± 0.1 and 1.1 ± 0.2 fold-change, respectively. The 100 mg/kg dose of SA most effectively boosted Nrf-2 and lowered Caspase-3 and IL-1β expression (Figure 6).

### 3.7 Molecular analysis of fibrogenic genes

The analysis of fibrosis-related genes (TGF-β and COL1A1) demonstrated significant changes across treatment groups. Control rats showed normalized baseline mRNA expression (1.0 ± 0.2 fold-change), which remained stable with SA alone treatment (p > 0.05).

TAA administration significantly upregulated fibrotic markers (p < 0.05): TGF-β increased by 125% (2.25 ± 0.2 fold-change) and COL1A1 by 225% (3.25 ± 0.4 fold-change), indicating substantial activation of pro-fibrotic pathways.

**FIGURE 7:**
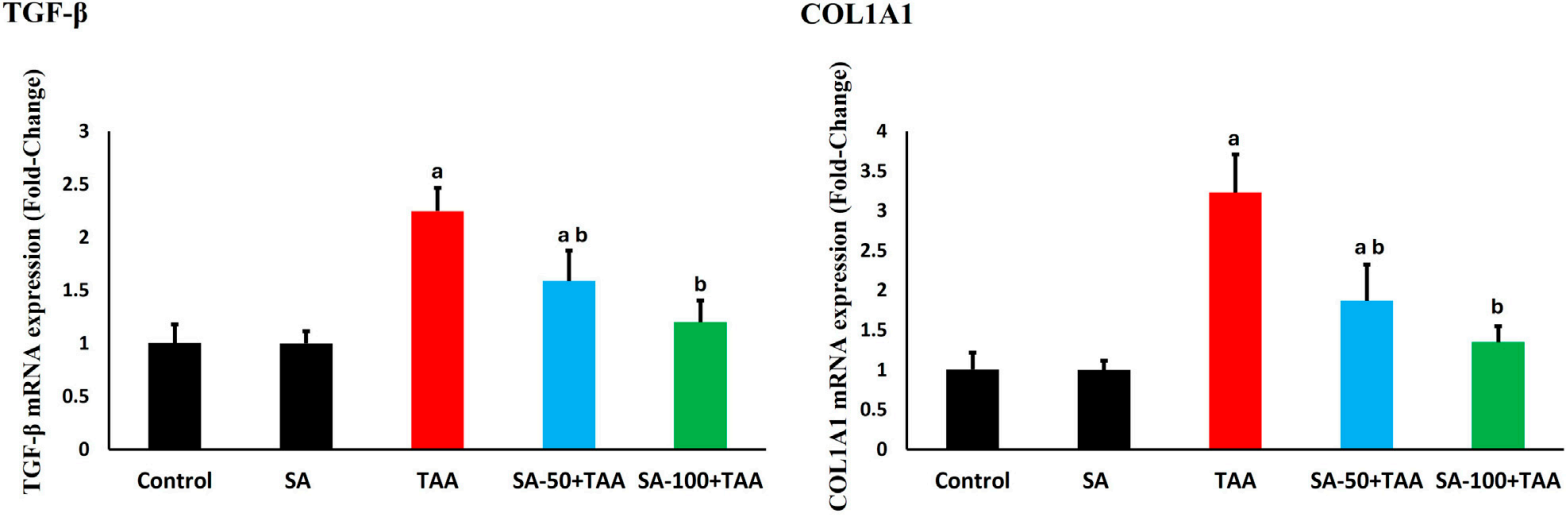
Effects of SA on hepatic mRNA expression levels of TGF-β and COL1A1 in TAA-induced liver fibrosis in rats. Values are expressed as mean ± SD (n = 10). ^a^ and ^b^ denote significant differences (p < 0.05) compared with the untreated control and TAA-injected groups, respectively.

SA co-treatment showed dose-dependent anti-fibrotic effects, with 100 mg demonstrating superior protection (p < 0.05): TGF-β expression decreased to 1.2 ± 0.2 fold-change and COL1A1 to 1.3 ± 0.2 fold-change. SA at the high dose most effectively suppressed TGF-β and COL1A1 expression (Figure 7).

### 3.8 Histological analysis

**FIGURE 8:**
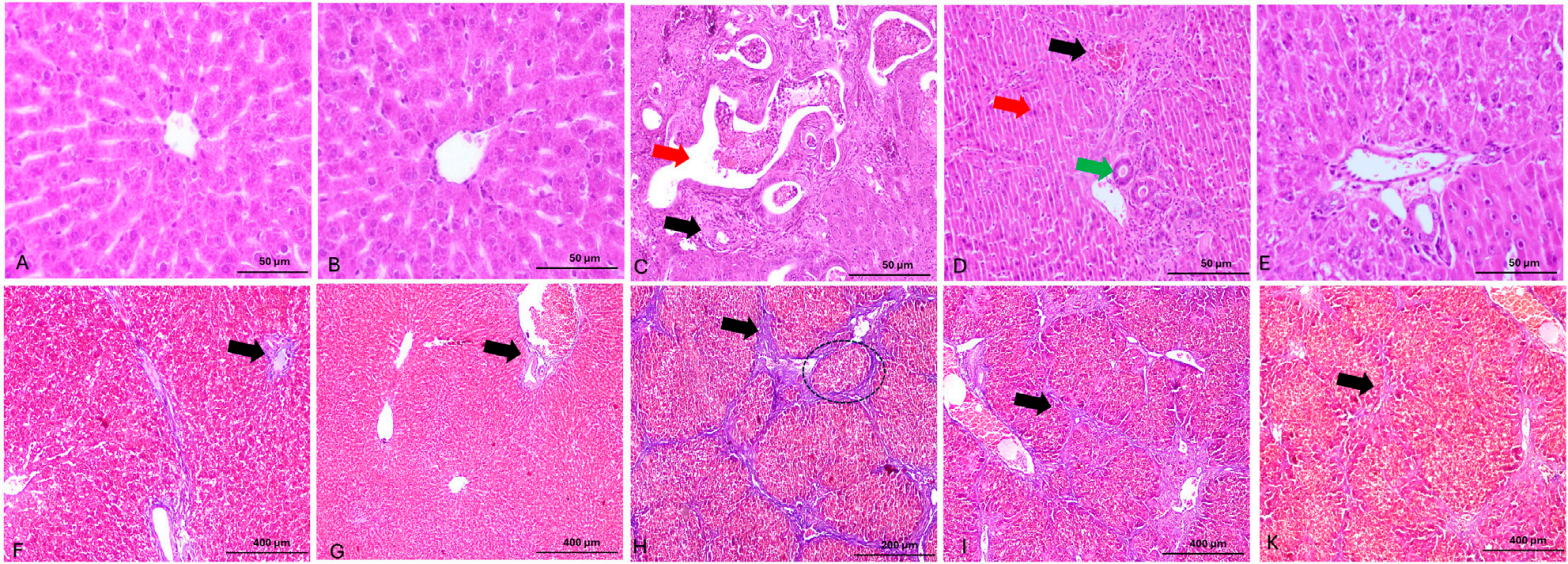
Panels **(A–E)** show representative photomicrographs of H&E-stained liver sections. **(A,B)** display normal hepatic architecture with well-organized hepatic cords radiating from central veins. **(C)** shows severe architectural disruption, with irregular ductal formations (green arrow) and portal vein branches exhibiting vascular remodeling and thickened walls (red arrow). **(D)** presents mild hepatocellular degeneration characterized by inflammatory cell infiltration and apoptosis (red arrow), increased bile duct profiles (green arrow), and hypertrophic biliary epithelium. **(E)** demonstrates moderate improvement in hepatic architecture, with partial resolution of inflammation and considerable preservation of normal liver structure. Panels **(F–K)** show Masson’s trichrome-stained liver sections. **(F,G)** exhibit minimal collagen deposition around portal areas and between hepatic septa (black arrow), indicating preserved architecture. **(H)** demonstrates extensive collagen accumulation forming bridging fibrosis (black arrow). **(I)** reveals moderate collagen deposition and fibrous septa formation (black arrow). **(K)** shows markedly reduced collagen content (black arrow) and restored hepatic architecture, reflecting the therapeutic effect of high-dose SA.

Histological analysis exhibited normal hepatic architecture characterized by well-organized hepatic cords radiating from central veins, with hepatocytes displaying normal polygonal morphology and distinct nuclei in control and SA-treated rats (Figures 8A,B), respectively. TAA administration resulted in severe hepatic fibrosis, characterized by extensive extracellular matrix deposition, formation of fibrous septa, and development of regenerative nodules. Histological examination revealed marked hepatocellular disorganization, proliferating ductal formations, parenchymal necrosis, inflammatory cell infiltration, and vascular distortion (Figure 8C). Co-administration of SA demonstrated significant dose-dependent hepatoprotective effects against TAA-induced injury. At lower doses, SA conferred moderate protection, evidenced by partial preservation of hepatic architecture and reduced inflammatory infiltration (Figure 8D). Higher dose administration exhibited enhanced therapeutic efficacy, characterized by marked preservation of hepatic parenchymal organization, significant attenuation of the inflammatory response, and maintenance of cellular integrity (Figure 8E).

These fibrotic changes were confirmed through Masson’s trichrome staining. Masson’s trichrome staining revealed differential collagen distribution patterns across experimental groups. Histological analysis exhibited minimal collagen deposition restricted to portal areas in control and SA-treated rats (Figures 8F,G), respectively. TAA administration induced severe fibrosis, evidenced by extensive collagen accumulation and blue-stained fibrous septa bridging portal and central venous structures, with prominent nodular formation (Figure 8C). Administration of SA (50 mg/kg) demonstrated notable reduction in collagen deposition and fibrous septae, with improved hepatic architectural preservation (Figure 8I). Higher dose intervention (100 mg/kg) showed superior anti-fibrotic effects, characterized by minimal collagen accumulation and significant preservation of parenchymal organization (Figure 8K).

## 4 Discussion

The present study demonstrates the hepatoprotective potential of SA against TAA-induced liver injury through multiple interconnected mechanisms, primarily involving modulation of oxidative stress, inflammation, and apoptotic pathways. Our findings reveal a complex network of protective mechanisms that collectively contribute to the therapeutic efficacy of SA.

The significant elevation in liver enzymes (ALP by 65%, AST by 114%, and ALT by 61%) following TAA administration indicates severe hepatocellular damage, consistent with findings by Shareef et al. (2023) and El-Demerdash et al. (2024), who reported similar enzymatic disruptions in chemical-induced liver injury. TAA elevation reflects compromised hepatocyte membrane integrity, leading to enzyme leakage into circulation. Notably, our results showed more severe AST elevation (90 ± 10) compared to El-Gendy et al. (2021), who reported a 51.93 ± 0.95 increase, suggesting potentially greater mitochondrial involvement in our model. Moreover, the pattern of enzyme elevation provides crucial insights into the nature and extent of hepatocellular damage (Kaur et al., 2019; El-Hameed et al., 2024). The pronounced elevation in AST/ALT ratio suggests significant mitochondrial involvement in the injury process, a finding that correlates with the observed oxidative stress parameters. TAA mitochondrial dysfunction likely contributes to the amplification of oxidative damage through increased electron leak and compromised ATP production (Chen et al., 2024). The ability of SA to normalize these enzyme levels suggests protection of both cellular and mitochondrial membranes, possibly through stabilization of membrane phospholipids and preservation of membrane-bound enzyme systems.

The oxidative stress parameters revealed a comprehensive disruption of cellular redox homeostasis by TAA. The severe depletion of GSH (67% reduction) represents a critical compromise of cellular antioxidant defense, exceeding the 40.6% reduction reported by Salam et al. (2013). TAA enhanced GSH depletion suggests a more severe oxidative challenge in our model. The concurrent elevation in NO (52%) and MDA (109%) levels indicates extensive oxidative damage and nitrosative stress, creating a self-perpetuating cycle of cellular injury.

SA demonstrated potent antioxidant activity by mitigating oxidative stress and restoring GSH levels. These results align with Manna et al. (2014), who reported that coconut water concentrate and its active constituent, SA, reversed H_2_O_2_-induced oxidative damage in hepatocytes by inhibiting NF-κB, activating the PI3K/Akt/Nrf2 pathway, and suppressing apoptosis via the SAPK/JNK/Bax axis. Furthermore, the interplay between oxidative stress markers reveals a complex cascade of cellular damage (Iqbal et al., 2024). The depletion of GSH not only compromises direct antioxidant defense but also affects multiple GSH-dependent detoxification pathways. The observed elevation in MDA levels indicates extensive lipid peroxidation, which can trigger a self-perpetuating cycle of membrane damage and cellular dysfunction. Notably, the ratio between GSH depletion and MDA elevation suggests a severe imbalance in cellular redox status that exceeds the compensatory capacity of endogenous antioxidant systems. SA ability to restore TAA balance indicates a comprehensive antioxidant mechanism that likely involves both direct radical scavenging and enhancement of cellular antioxidant capacity.

The comprehensive impairment of antioxidant enzymes by TAA (CAT ↓63%, GPx ↓58%, GR ↓50%, SOD ↓45%) reveals a systematic breakdown of cellular antioxidant defenses. These findings align with those reported by Jorgačević et al. (2022), who observed that TAA-induced nephrotoxicity increases SOD and CAT activity, as well as GSH levels. The dose-dependent restoration of these enzymes by SA, particularly at 100 mg, demonstrates a coordinated enhancement of antioxidant defense systems.

The differential effects on various antioxidant enzymes provide insights into the hierarchy of oxidative damage. The more severe reduction in CAT activity compared to other enzymes suggests particular vulnerability of peroxide detoxification pathways. The coordinated restoration of enzyme activities by SA indicates a systematic enhancement of antioxidant defense that may involve both transcriptional activation and post-translational stabilization of enzyme systems. TAA comprehensive enzyme modulation distinguishes SA from other antioxidant compounds that typically show more selective effects on specific enzymes.

At the molecular level, TAA administration led to a suppression of Nrf2 mRNA expression. In contrast, our results demonstrated that SA potently activated the Nrf2 pathway (1.7 ± 0.2 fold-change). TAA finding aligns with Manna et al. (2014), who reported significant activation of the transcription factor Nrf2 when treating hydroperoxide-induced oxidative damage in hepatocytes with coconut water concentrate containing SA as its active component. The enhanced Nrf2 activation represents a crucial molecular mechanism underlying the observed antioxidant effects, as Nrf2 regulates the transcription of multiple antioxidant enzymes. The temporal relationship between Nrf2 activation and subsequent antioxidant enzyme upregulation suggests a coordinated cellular stress response. The enhanced Nrf2 nuclear translocation observed in our study indicates efficient sensing of oxidative stress and rapid activation of protective pathways. TAA heightened Nrf2 response may explain the superior antioxidant enzyme modulation observed with SA treatment. Moreover, the sustained activation of Nrf2 suggests long-term adaptation of cellular antioxidant capacity rather than merely acute stress response.

The hierarchical organization of protective pathways reveals sophisticated regulation of cellular defense mechanisms. The primary activation of Nrf2 initiates a cascade of protective events that appear to be temporally coordinated. The rapid induction of antioxidant enzymes is followed by sustained modulation of inflammatory and fibrogenic pathways, suggesting that the antioxidant effects of SA serve as a master regulator of downstream protective mechanisms.

The inflammatory response to TAA administration was characterized by significant elevations in key pro-inflammatory mediators: TNF-α (76%), IL-1β (67%), and NF-κB (95%). The increase in IL-1β was confirmed at the transcriptional level through elevated mRNA expression, demonstrating regulation at both protein and gene levels. Zhao et al. (2021) similarly showed that dihydromyricetin inhibited NF-κB activation and reduced both TNF-α and IL-1β levels in TAA-treated mice, underscoring the central role of NF-κB–mediated inflammation in fibrogenesis, supporting the role of inflammatory cascade activation in the pathogenesis of hepatic injury. However, SA ability to normalize these inflammatory markers, particularly at 100 mg, demonstrates anti-inflammatory properties that complement its antioxidant effects.

The relationship between oxidative stress and inflammation in our model demonstrates a complex bidirectional interaction. The elevation in pro-inflammatory cytokines coincides with peaks in oxidative stress markers, suggesting that these pathways amplify each other’s effects (Kumar et al., 2012). The ability of SA to suppress both pathways simultaneously indicates interference with TAA self-perpetuating cycle. Notably, the reduction in NF-κB levels (95% to near baseline) suggests that SA may directly modulate inflammatory signaling pathways in addition to its antioxidant effects. TAA dual action potentially explains the superior anti-inflammatory effects observed in our study compared to conventional antioxidants. The temporal pattern of inflammatory marker reduction correlates strongly with the restoration of antioxidant capacity, suggesting that the anti-inflammatory effects may be secondary to oxidative stress reduction.

We observed that TAA-induced liver fibrosis was associated with increased pro-apoptotic protein expression (Bax and caspase-3), in addition to a decrease in anti-apoptotic protein expression (Bcl-2). This mirrors Eissa et al. (2018), who observed a 2.2-fold increase in caspase-3 mRNA after TAA exposure, and (Mousa et al., 2019), who showed that Moringa oleifera extract downregulated caspase-3 protein in TAA-treated rats. Importantly, our 100 mg/kg SA treatment brought Bax, caspase-3, and Bcl-2 levels back toward control values—providing precise protein-level confirmation that correcting apoptotic dysregulation is a central mechanism of SA’s protective effect.

The ability of SA to normalize these parameters suggests a comprehensive cytoprotective effect. The modulation of apoptotic pathways by SA reveals sophisticated regulation of cell death mechanisms. The observed changes in Bax/B-cell lymphoma-2 (Bcl2) ratio suggest that oxidative stress-induced apoptosis is a major mechanism of cell death in our model (Warren et al., 2000). The ability of SA to normalize TAA ratio while simultaneously reducing caspase-3 levels indicates protection at multiple points in the apoptotic cascade. The correlation between reduced oxidative stress markers and normalized apoptotic proteins suggests that SA anti-apoptotic effects are mediated primarily through its antioxidant properties. TAA protection extends to mitochondrial pathway regulation, as evidenced by the preservation of mitochondrial membrane potential and reduced cytochrome c release, effects not previously reported with similar compounds (Rodrigues et al., 1999; Petrosillo et al., 2004).

The significant upregulation of fibrotic markers (TGF-β↑125%, COL1A1↑225%) by TAA demonstrates robust activation of pro-fibrotic pathways. These results are consistent with Zeweil et al. (2020), who demonstrated that TAA administration (200 mg/kg) for 15 weeks induced liver fibrosis in rats, characterized by increased hepatic mRNA expression of TGF-β and COL1A1 compared to control rats. The superior anti-fibrotic effects of SA, particularly at 100 mg, suggest effective interruption of fibrogenic signaling cascades.

The relationship between oxidative stress and fibrogenesis in our model provides new insights into the mechanisms of hepatic fibrosis. The marked elevation in TGF-β and COL1A1 expression correlates strongly with oxidative stress markers, suggesting that redox disturbance is a key driver of fibrogenic activation. SA ability to suppress these fibrotic markers while restoring antioxidant capacity indicates that its anti-fibrotic effects may be primarily mediated through oxidative stress reduction. The observed decrease in myofibroblast activation and reduced stellate cell proliferation suggest additional mechanisms beyond direct antioxidant effects. The temporal sequence of these changes indicates that early oxidative stress reduction may prevent the initiation of fibrogenic pathways rather than merely suppressing established fibrosis.

The histological findings correlate well with the molecular and biochemical parameters, showing severe tissue architecture disruption by TAA and significant protection by SA. These observations provide structural validation of the biochemical findings and demonstrate the translation of molecular effects to tissue-level protection.

The histological findings provide crucial validation of the molecular and biochemical observations. The preservation of hepatic architecture in SA-treated groups correlates strongly with reduced oxidative stress markers and improved antioxidant capacity. The reduced inflammatory infiltration and decreased collagen deposition observed microscopically align with the molecular markers of inflammation and fibrosis. Particularly noteworthy is the preservation of sinusoidal structure and reduced stellate cell activation in treated groups, suggesting that SA antioxidant effects extend to maintaining microvascular integrity. The dose-dependent improvement in histological parameters provides visual confirmation of the biochemical findings and supports the therapeutic potential of SA in liver injury.

SA exerts its hepatoprotective and antifibrotic effects against TAA-induced liver fibrosis through multiple interconnected mechanisms. Primarily, it activates the Nrf2 signaling pathway, leading to the upregulation of antioxidant enzymes such as SOD, CAT, GPx, and GR, which collectively reduce oxidative stress. In parallel, it suppresses the NF-κB pathway, resulting in the downregulation of pro-inflammatory cytokines like TNF-α and IL-1β. Furthermore, SA modulates the expression of apoptotic markers (e.g., Bax, Bcl-2, and caspase-3), thereby preventing hepatocyte apoptosis. Additionally, it inhibits the expression of fibrogenic genes such as TGF-β1 and COL1A1, which are critical drivers of extracellular matrix deposition and fibrosis. These combined effects contribute to the overall protective role of SA in TAA-induced hepatic injury.

## 5 Conclusion

In this study, we demonstrate that SA significantly attenuates TAA-induced hepatic fibrosis in rats by activating the Nrf2 antioxidant pathway and suppressing NF-κB–mediated inflammatory signaling. Over the 6-week treatment period (50 and 100 mg/kg, i. p., daily), SA was well tolerated, with no observable signs of toxicity or behavioral changes at either dose. SA treatment reduced oxidative-stress markers, downregulated pro-inflammatory cytokines, inhibited hepatocyte apoptosis, and decreased expression of fibrogenic genes, collectively leading to reduced collagen deposition and improved liver histology. Although no adverse effects were noted at the doses tested, further studies are warranted to define the safety profile of higher doses and longer treatment durations. These findings highlight the therapeutic potential of SA as an effective antifibrotic agent, while underscoring the need for additional work on optimal dosing and treatment length.

## Data Availability

The raw data supporting the conclusions of this article will be made available by the authors, without undue reservation.

## References

[B1] Aebi H. (1984). “[13] catalase *in vitro* ,” in Methods in enzymology (Elsevier), 121–126. 10.1016/s0076-6879(84)05016-3 6727660

[B2] Ahmad N. Asghar S. Gillani S. A. (2022). Transfer learning-assisted multi-resolution breast cancer histopathological images classification. Vis. Comput. 38, 2751–2770. 10.1007/s00371-021-02153-y

[B3] Al-Malki A. L. (2019). Shikimic acid from Artemisia absinthium inhibits protein glycation in diabetic rats. Int. J. Biol. Macromol. 122, 1212–1216. 10.1016/j.ijbiomac.2018.09.072 30227208

[B4] Bao X. Zheng Z. Lv J. Bao J. Chang S. Jiang X. (2023). Shikimic acid (SA) inhibits neuro-inflammation and exerts neuroprotective effects in an LPS-induced *in vitro* and *in vivo* model. Front. Pharmacol. 14, 1265571. 10.3389/fphar.2023.1265571 38026972 PMC10652795

[B5] Bardallo G. Panisello‐Roselló A. Sanchez‐Nuno S. Alva N. Roselló‐Catafau J. Carbonell T. (2022). Nrf2 and oxidative stress in liver ischemia/reperfusion injury. FEBS J. 289, 5463–5479. 10.1111/febs.16336 34967991

[B6] Candeias N. R. Assoah B. Simeonov S. P. (2018). Production and synthetic modifications of shikimic acid. Chem. Rev. 118, 10458–10550. 10.1021/acs.chemrev.8b00350 30350584

[B7] Cardiff R. D. Miller C. H. Munn R. J. (2014). Manual hematoxylin and eosin staining of mouse tissue sections. Cold Spring Harb. Protoc. 2014, 655–658. pdb. prot073411. 10.1101/pdb.prot073411 24890205

[B8] Carmo De Carvalho E Martins M. D. Da Silva Santos Oliveira A. S. Da Silva L. a.A. Primo M. G. S. De Carvalho Lira V. B. (2022). “Biological indicators of oxidative stress [malondialdehyde, catalase, glutathione peroxidase, and superoxide dismutase] and their application in nutrition,” in Biomarkers in nutrition (Springer), 1–25.

[B9] Che R. Yuan Y. Huang S. Zhang A. (2014). Mitochondrial dysfunction in the pathophysiology of renal diseases. Am. J. Physiology-Renal Physiology 306, F367–F378. 10.1152/ajprenal.00571.2013 24305473

[B10] Chen P. Yao L. Yuan M. Wang Z. Zhang Q. Jiang Y. (2024). Mitochondrial dysfunction: a promising therapeutic target for liver diseases. Genes and Dis. 11, 101115. 10.1016/j.gendis.2023.101115 PMC10828599 38299199

[B11] Drury R. Wallington E. (1980). Carleton’s histological technique. 5th ed., 270. New York: Churchill Livingstone.

[B12] Dwivedi D. K. Jena G. (2022). Simultaneous modulation of NLRP3 inflammasome and Nrf2/ARE pathway rescues thioacetamide-induced hepatic damage in mice: role of oxidative stress and inflammation. Inflammation 45, 610–626. 10.1007/s10753-021-01571-3 34664134

[B13] Eissa L. A. Kenawy H. I. El-Karef A. Elsherbiny N. M. El-Mihi K. A. (2018). Antioxidant and anti-inflammatory activities of berberine attenuate hepatic fibrosis induced by thioacetamide injection in rats. Chemico-biological Interact. 294, 91–100. 10.1016/j.cbi.2018.08.016 30138605

[B14] El-Demerdash F. M. Al Mhanna A. B. El-Sayed R. A. Mohamed T. M. Salem M. M. (2024). Hepatoprotective impact of Nigella sativa silver nanocomposite against genotoxicity, oxidative stress, and inflammation induced by thioacetamide. Tissue Cell 87, 102332. 10.1016/j.tice.2024.102332 38367325

[B15] El-Gendy Z. A. El-Marasy S. A. Ahmed R. F. El-Batran S. A. Abd El-Rahman S. S. Ramadan A. (2021). Hepatoprotective effect of Saccharomyces Cervisciae Cell Wall Extract against thioacetamide-induced liver fibrosis in rats. Heliyon 7, e07159. 10.1016/j.heliyon.2021.e07159 34159266 PMC8203708

[B16] El-Hameed S. A. Ibrahim I. Awadin W. El-Shaieb A. (2024). Assessment of single and combined administration of ubiquinone and lactoferrin on histopathology, ultrastructure, oxidative stress, and WNT4 expression gene induced by thioacetamide on hepatorenal system of adult male rats. Beni-Suef Univ. J. Basic Appl. Sci. 13, 41. 10.1186/s43088-024-00494-w

[B17] Ellman G. L. (1959). Tissue sulfhydryl groups. Archives Biochem. biophysics 82, 70–77. 10.1016/0003-9861(59)90090-6 13650640

[B18] Estevez M. Estevez J. (2012). A short overview on the medicinal chemistry of (—)-shikimic acid. Mini Rev. Med. Chem. 12, 1443–1454. 10.2174/138955712803832735 22827174

[B19] Ezhilarasan D. (2023). Molecular mechanisms in thioacetamide-induced acute and chronic liver injury models. Environ. Toxicol. Pharmacol. 99, 104093. 10.1016/j.etap.2023.104093 36870405

[B20] Gandhi G. R. Vasconcelos A. B. S. Antony P. J. Montalvão M. M. De Franca M. N. F. Hillary V. E. (2023). Natural sources, biosynthesis, biological functions, and molecular mechanisms of shikimic acid and its derivatives. Asian Pac. J. Trop. Biomed. 13, 139–147. 10.4103/2221-1691.374230

[B21] Garvey W. (1984). Modified elastic tissue-Masson trichrome stain. Stain Technol. 59, 213–216. 10.3109/10520298409113858 6208643

[B22] Gu Y. Zhang J. Zheng H. Qin Y. Zheng M. Hu Y. (2024). Therapeutic effect of shikimic acid on heat stress-induced myocardial damage: assessment via network pharmacology, molecular docking, molecular dynamics simulation, and *in vitro* experiments. Pharmaceuticals 17, 1485. 10.3390/ph17111485 39598396 PMC11597562

[B23] Iqbal M. J. Kabeer A. Abbas Z. Siddiqui H. A. Calina D. Sharifi-Rad J. (2024). Interplay of oxidative stress, cellular communication and signaling pathways in cancer. Cell Commun. Signal. 22, 7. 10.1186/s12964-023-01398-5 38167159 PMC10763046

[B24] Jomova K. Alomar S. Y. Alwasel S. H. Nepovimova E. Kuca K. Valko M. (2024). Several lines of antioxidant defense against oxidative stress: antioxidant enzymes, nanomaterials with multiple enzyme-mimicking activities, and low-molecular-weight antioxidants. Archives Toxicol. 98, 1323–1367. 10.1007/s00204-024-03696-4 PMC11303474 38483584

[B25] Jorgačević B. Stanković S. Filipović J. Samardžić J. Vučević D. Radosavljević T. (2022). Betaine modulating MIF-mediated oxidative stress, inflammation and fibrogenesis in thioacetamide-induced nephrotoxicity. Curr. Med. Chem. 29, 5254–5267. 10.2174/0929867329666220408102856 35400322

[B26] Kaur S. Sharma D. Singh A. P. Kaur S. (2019). Amelioration of hepatic function, oxidative stress, and histopathologic damages by Cassia fistula L. fraction in thioacetamide-induced liver toxicity. Environ. Sci. Pollut. Res. 26, 29930–29945. 10.1007/s11356-019-06158-y 31407268

[B27] Kirkland R. A. Franklin J. L. (2003). Bax, reactive oxygen, and cytochrome c release in neuronal apoptosis. Antioxidants Redox Signal. 5, 589–596. 10.1089/152308603770310257 14580315

[B28] Kumar R. Prakash S. Chhabra S. Singla V. Madan K. Gupta S. D. (2012). Association of pro-inflammatory cytokines, adipokines and oxidative stress with insulin resistance and non-alcoholic fatty liver disease. Indian J. Med. Res. 136, 229–236. 10.4103/0971-5916.98879 22960889 PMC3461734

[B29] Lee J. Nguyen Q. N. Park J. Y. Lee S. Hwang G. S. Yamabe N. (2020). Protective effect of shikimic acid against cisplatin-induced renal injury: *in vitro* and *in vivo* studies. Plants 9, 1681. 10.3390/plants9121681 33271750 PMC7759863

[B30] Li S. Hong M. Tan H.-Y. Wang N. Feng Y. (2016). Insights into the role and interdependence of oxidative stress and inflammation in liver diseases. Oxidative Med. Cell. Longev. 2016, 4234061. 10.1155/2016/4234061 PMC5192343 28070230

[B31] Lowry O. H. Rosebrough N. J. Farr A. L. Randall R. J. (1951). Protein measurement with the Folin phenol reagent. J. Biol. Chem. 193, 265–275. 10.1016/s0021-9258(19)52451-6 14907713

[B32] Manna K. Khan A. Das D. K. Kesh S. B. Das U. Ghosh S. (2014). Protective effect of coconut water concentrate and its active component shikimic acid against hydroperoxide mediated oxidative stress through suppression of NF-κB and activation of Nrf2 pathway. J. Ethnopharmacol. 155, 132–146. 10.1016/j.jep.2014.04.046 24835026

[B33] Moron M. S. Depierre J. W. Mannervik B. (1979). Levels of glutathione, glutathione reductase and glutathione S-transferase activities in rat lung and liver. Biochimica biophysica acta (BBA)-general Subj. 582, 67–78. 10.1016/0304-4165(79)90289-7 760819

[B34] Mousa A. A. El-Gansh H. a.I. Eldaim M. a.A. Mohamed M. a.E. Morsi A. H. El Sabagh H. S. (2019). Protective effect of Moringa oleifera leaves ethanolic extract against thioacetamide-induced hepatotoxicity in rats via modulation of cellular antioxidant, apoptotic and inflammatory markers. Environ. Sci. Pollut. Res. Int. 26, 32488–32504. 10.1007/s11356-019-06368-4 31617137

[B36] Nishikimi M. Rao N. A. Yagi K. (1972). The occurrence of superoxide anion in the reaction of reduced phenazine methosulfate and molecular oxygen. Biochem. biophysical Res. Commun. 46, 849–854. 10.1016/s0006-291x(72)80218-3 4400444

[B37] Ohkawa H. Ohishi N. Yagi K. (1979). Assay for lipid peroxides in animal tissues by thiobarbituric acid reaction. Anal. Biochem. 95, 351–358. 10.1016/0003-2697(79)90738-3 36810

[B38] Paglia D. E. Valentine W. N. (1967). Studies on the quantitative and qualitative characterization of erythrocyte glutathione peroxidase. J. laboratory Clin. Med. 70, 158–169. 10.5555/uri:pii:0022214367900765 6066618

[B54] Pfaffl M. W. (2001). A new mathematical model for relative quantification in real-time RTPCR. Nucleic Acids Res. 29 (9), e45. 10.1093/nar/29.9.e45 PMC55695 11328886

[B39] Petrosillo G. Ruggiero F. M. Pistolese M. Paradies G. (2004). Ca2+-induced reactive oxygen species production promotes cytochrome c release from rat liver mitochondria via mitochondrial permeability transition (MPT)-dependent and MPT-independent mechanisms: role of cardiolipin. J. Biol. Chem. 279, 53103–53108. 10.1074/jbc.M407500200 15475362

[B40] Poynard T. Bedossa P. Opolon P. (1997). Natural history of liver fibrosis progression in patients with chronic hepatitis C. The OBSVIRC, METAVIR, CLINIVIR, and DOSVIRC groups. Lancet 349, 825–832. 10.1016/s0140-6736(96)07642-8 9121257

[B41] Quiroz D. C. D. Carmona S. B. Bolívar F. Escalante A. (2014). Current perspectives on applications of shikimic and aminoshikimic acids in pharmaceutical chemistry. Res. Rep. Med. Chem., 35–46. 10.2147/rrmc.s46560

[B42] Reitman S. Frankel S. (1957). A colorimetric method for the determination of serum glutamic oxalacetic and glutamic pyruvic transaminases. Am. J. Clin. pathology 28, 56–63. 10.1093/ajcp/28.1.56 13458125

[B43] Robbins R. A. Floreani A. A. Von Essen S. G. Sisson J. H. Hill G. E. Rubinstein I. (1996). Measurement of exhaled nitric oxide by three different techniques. Am. J. Respir. Crit. care Med. 153, 1631–1635. 10.1164/ajrccm.153.5.8630613 8630613

[B44] Rodrigues C. M. Ma X. Linehan-Stieers C. Fan G. Kren B. T. Steer C. J. (1999). Ursodeoxycholic acid prevents cytochrome c release in apoptosis by inhibiting mitochondrial membrane depolarization and channel formation. Cell Death and Differ. 6, 842–854. 10.1038/sj.cdd.4400560 10510466

[B45] Roufayel R. (2016). Regulation of stressed-induced cell death by the Bcl-2 family of apoptotic proteins. Mol. Membr. Biol. 33, 89–99. 10.1080/09687688.2017.1400600 29166806

[B46] Salam O. A. Mohammed N. Sleem A. Farrag A. (2013). The effect of antidepressant drugs on thioacetamide-induced oxidative stress. Eur. Rev. Med. and Pharmacol. Sci. 17, 735–744. 23609356

[B35] Salguero Palacios R. Roderfeld M. Hemmann S. Rath T. Atanasova S. Tschuschner A. (2008). Activation of hepatic stellate cells is associated with cytokine expression in thioacetamide-induced hepatic fibrosis in mice. Laboratory Investigation. 88(11), 1192–1203. 10.1038/labinvest.2008.91 18794850

[B47] Santos-Sánchez N. F. Salas-Coronado R. Hernández-Carlos B. Villanueva-Cañongo C. (2019). Shikimic acid pathway in biosynthesis of phenolic compounds. Plant physiological aspects phenolic Compd. 1, 1–15. 10.5772/intechopen.85947

[B48] Shareef S. H. Al-Medhtiy M. H. Al Rashdi A. S. Aziz P. Y. Abdulla M. A. (2023). Hepatoprotective effect of pinostrobin against thioacetamide-induced liver cirrhosis in rats. Saudi J. Biol. Sci. 30, 103506. 10.1016/j.sjbs.2022.103506 36458098 PMC9706172

[B49] Sun J.-Y. You C.-Y. Dong K. You H.-S. Xing J.-F. (2016). Anti-inflammatory, analgesic and antioxidant activities of 3, 4-oxo-isopropylidene-shikimic acid. Pharm. Biol. 54, 2282–2287. 10.3109/13880209.2016.1153663 27609150

[B50] Warren M. C. Bump E. A. Medeiros D. Braunhut S. J. (2000). Oxidative stress–induced apoptosis of endothelial cells. Free Radic. Biol. Med. 29, 537–547. 10.1016/s0891-5849(00)00353-1 11025197

[B51] Xing J. You C. Dong K. Sun J. You H. Dong Y. (2013). Ameliorative effects of 3, 4-oxo-isopropylidene-shikimic acid on experimental colitis and their mechanisms in rats. Int. Immunopharmacol. 15, 524–531. 10.1016/j.intimp.2013.02.008 23434856

[B52] Zeweil M. M. Sadek K. M. Elsadek M. F. Mahmoud S. F. Ahmed B. M. Khafaga A. F. (2020). Sidr honey abrogates the oxidative stress and downregulates the hyaluronic acid concentration and gene expression of TGF‐β1 and COL1a1 in rat model of thioacetamide‐induced hepatic fibrosis. Animal Sci. J. 91, e13434. 10.1111/asj.13434 32696560

[B53] Zhao Y. Liu X. Ding C. Gu Y. Liu W. (2021). Dihydromyricetin reverses thioacetamide-induced liver fibrosis through inhibiting NF-κB-mediated inflammation and TGF-β1-regulated of PI3K/Akt signaling pathway. Front. Pharmacol. 12, 783886. 10.3389/fphar.2021.783886 34867416 PMC8634482

